# Social mobility and health gain: the combined effects of material conditions, psychological support, and social capital

**DOI:** 10.3389/fpubh.2025.1496279

**Published:** 2025-03-12

**Authors:** Lu Zhang, Hai Gu, Huiying Chen, Qinglin Xu, Zi Lin, Yang Yi

**Affiliations:** ^1^Center for Health Policy and Management Studies, School of Government, Nanjing University, Nanjing, Jiangsu Province, China; ^2^School of Business, Anyang Normal University, Anyang, Henan Province, China

**Keywords:** social mobility, self-rated health, material conditions, psychological support, social capital

## Abstract

**Rationale:**

Research on how social mobility impacts health has primarily focused on developed countries or regions, with a notable absence of in-depth examination into the underlying mechanisms responsible for these influences.

**Objective:**

This paper utilizes data from the 2021 Chinese General Social Survey to focus on the health effects of social mobility in China and the underlying mechanisms behind these effects.

**Methods:**

We employed an ordered logistic regression model as the baseline to test the health effects of social mobility. To address endogeneity issues, we used placebo tests, instrumental variable methods, and the Karlson-Holm-Breen mediation analysis to explore the pathways through which social mobility affects health.

**Results:**

Our findings indicate that upward social mobility is associated with better self-rated health, and this conclusion holds in China. The health benefits of upward social mobility are more pronounced for males and individuals with lower initial socioeconomic status. Upward social mobility primarily influences individuals’ health through material conditions, psychological support, and social capital.

**Conclusion:**

Our research findings support the rising from rags hypothesis, expanding the research context of social mobility theory, which provides a new perspective on promoting health equity and improving health within the life course context.

## 1 Introduction

Since the founding of the People’s Republic of China, per capita disposable income has risen from 49.7 yuan to 35,128.1 yuan by 2021, and life expectancy has increased from less than 35 years at the time of the country’s establishment to 77.93 years according to the seventh national census. In terms of efficiency, the People’s Republic of China has undoubtedly presented a commendable track record to the world in enhancing living standards and improving overall health conditions. However, we must also acknowledge the shortcomings in terms of equity: China has a large population that is rapidly aging, and there remains a significant disparity in health status between the wealthy and the poor, as well as across different social classes. There are notable examples of “pro-rich” and “pro-urban”^1^ health inequalities.

Policies that tackle health inequalities should not only focus on facilitating more equitable access to healthcare services but also emphasize interventions on the root causes, specifically the social determinants of health (1). Social causation theory posits that socioeconomic status (SES) constitutes a structural determinant influencing individual health, with health outcomes resulting from socioeconomic deprivation. Systematic disparities in health outcomes among different socioeconomic strata have been amply documented in research, with evidence across nations consistently demonstrating a gradient effect between SES and health. Populations disadvantaged socioeconomically experience higher morbidity and mortality rates, while those with higher SES enjoy longer and healthier lives compared to their lower-SES counterparts (2). Socioeconomic status is considered a fundamental factor shaping health levels and is one of the primary contributors to health inequalities. Overcoming the negative impact of disadvantaged socioeconomic environments on residents’ health and breaking free from the health inequalities caused by socioeconomic factors have become urgent challenges that countries worldwide need to address.

The critical period theory posits that health inequalities stem from risk exposures during key developmental stages, which can produce lasting and irreversible “scarring effects” on health (3). The cumulative disadvantage theory emphasizes that health disparities result from the gradual accumulation of all health risk factors experienced earlier in life, based on the frequency, duration, and severity of exposure (4). In contrast, the social mobility theory offers a new perspective. It measures social mobility through changes in education, income, and social prestige, focusing on how such mobility influences health outcomes (5, 6). This theory underscores the malleability and reversibility of adverse socioeconomic impacts (7), suggesting that improving later-life environments or achieving social class transitions can partially or fully mitigate the negative health effects of early socioeconomic disadvantages. However, can social mobility theory be tested in practice? Does upward social mobility improve health outcomes? What are the micro-mechanisms through which social mobility affects health and well-being? Can current policies improve health outcomes by promoting social mobility, and which groups should be prioritized in this process? Clarifying these issues will significantly affect the development of theoretical frameworks and public health policies.

This paper endeavors to address the above questions. The contributions of this study are: First, current research on the health effects of social mobility is relatively limited, and most related studies are concentrated in developed countries such as the United States or Europe, with fewer studies focusing on developing countries or regions. Since the founding of the People’s Republic of China, rapid economic growth and the expansion of higher education have created unprecedented opportunities for social mobility, making China a natural “experimental field” for studying social mobility. Based on the social background with Chinese characteristics, this study expands the applicable scenarios and research perspectives of the social mobility theory. Second, diverging from prior research that primarily focuses on whether mobility occurs and the directional health effects without delving deeply into the underlying mechanisms, this paper elucidates the micro-level mechanisms through which social class ascension influences health, examining its impact via three dimensions: material conditions, psychological support, and social capital, which provides insights for improving health and promoting equal health opportunities in the context of social mobility. Third, adopting a heterogeneity lens, this study elucidates the advantages or limitations of health effects attributable to social mobility across different population segments. It charts a direction for identifying vulnerable groups amidst social class transformation while outlines a pathway for realizing health equity and maximizing the efficacy of health resource allocation.

## 2 Theory and hypotheses

Dominant theoretical perspectives in the extant literature on the health effects of social mobility can be synthesized into three main categories: the harmful mobility theory, the beneficial mobility theory, and the neutral theory.

### 2.1 Theory

#### 2.1.1 The harmful mobility theory

The dissociative hypothesis depicts individuals who achieve social mobility as “socially isolated individuals caught between the margins, experiencing dual exclusion from both their origin and destination social classes” (8). It suggests that any form of social mobility may have adverse effects on health. From a psychological health perspective, individuals experiencing social mobility must adapt to the norms of a new social group, a process often accompanied by stress, which can lead to feelings of isolation and alienation, potentially resulting in anxiety and depression (9). From a physical health standpoint, prolonged psychological stress may activate the stress response system, increasing the risk of cardiovascular diseases and immune suppression (10). The frustrated achiever hypothesis further posits that new entrants are at a disadvantage compared to the original members of their destination class. These achievers may compare their situation to those stable in higher classes, realize their relatively lower status, and consequently feel negative emotions, which can adversely affect their health and well-being (11). Early stress theories and clinical studies have also identified the negative impacts of social mobility on mental and physical health, showing that class transitions can deteriorate the psychological health or well-being of some individuals. This conclusion has been validated in European countries such as Germany, the United Kingdom, and Poland (12–14).

Existing research generally concurs that downward social mobility exerts a detrimental impact on health. For instance, the ‘falling from grace’ thesis posits that such mobility, entailing a loss, often involves involuntary and uncontrollable negative life events such as bankruptcy, unemployment, or divorce, which signify a status, power, or income decline. Individuals may struggle to adapt to their new, deteriorated living circumstances (15). Moreover, downward mobility can be perceived as a failure to meet societal expectations or personal aspirations, potentially giving rise to negative emotions such as self-blame, depression, pessimism, fear, and even anger and feelings of injustice, which can leave lasting scars on an individual’s health (16). Gugushvili and Prag (17) further illustrate that the health toll of downward mobility on participants manifests in increased substance abuse, smoking, alcohol and drug consumption, depression, anxiety, fear, weight gain, exacerbation or onset of chronic diseases, among other health indicators (18).

#### 2.1.2 The beneficial mobility theory

The rising from rags hypothesis, originating from social psychology research, suggests that the positive impacts of upward social mobility on health outweigh the potential negatives (19). Enabled by the fulfillment of socioeconomic aspirations and enhanced feelings of personal control, upward social mobility is seen as an aspirational process where overcoming adversity to escape disadvantageous socioeconomic positions generates satisfaction and a sense of achievement that can buffer against psychosocial stressors during the mobility process (20). Moreover, it fosters gratitude and endorsement toward the societal structures facilitating the upward leap (21), positively influencing health. Studies have revealed that individuals experiencing upward social mobility exhibit reduced mortality rates (22), lower morbidity (23), improved cognitive function (24), lower depression scores (25), fewer functional impairments (26), better self-rated health (27), heightened happiness, and healthier behaviors (28). Compared to those experiencing downward mobility, upwardly mobile individuals demonstrate superior physical and mental health and higher levels of well-being (29).

#### 2.1.3 The neutral theory

The acculturation hypothesis frames social mobility as a process of cultural adaptation to the normative values and lifestyles associated with the destination social status, a matter of assimilation or resocialization, devoid of the psychological distress implied by “separation” (30). Through the lens of acculturation theory, the impact of social mobility on individual health and well-being is largely contingent upon the degree to which individuals integrate into their new class milieu; the lower the level of social integration, the poorer the health outcomes. Hence, the health effects of social mobility are also mediated by the discrepancy between the origin and destination social classes, with both the origin and destination exerting some influence on those undergoing mobility. According to Houle (31), the destination class exerts a stronger influence on health status than the origin class, given the closer relationship between current class position and immediate outcomes. In summary, the acculturation hypothesis emphasizes that the impact of social mobility on health largely depends on the level of integration achieved and the class of origin and destination, with the destination class potentially having a more significant impact on health outcomes.

#### 2.1.4 Limitations of current research

Existing research exhibits several limitations, mainly in the following areas: Firstly, from the perspective of research subjects, the current studies on the health effects of social mobility are relatively limited, with the majority focusing on developed countries or regions, while research on developing countries or areas is scarce. Secondly, from the perspective of research content, existing studies mostly remain at the superficial level of examining whether social mobility affects health status, lacking in-depth exploration of how social mobility influences health status. Thirdly, from the perspective of variable measurement methods, existing empirical research predominantly characterizes social mobility through changes in objective socioeconomic status indicators such as education, income, and occupation. However, studies incorporating subjective indicators, such as self-perceived social mobility, still need further enrichment.

### 2.2 Hypotheses

**Figure 1:**
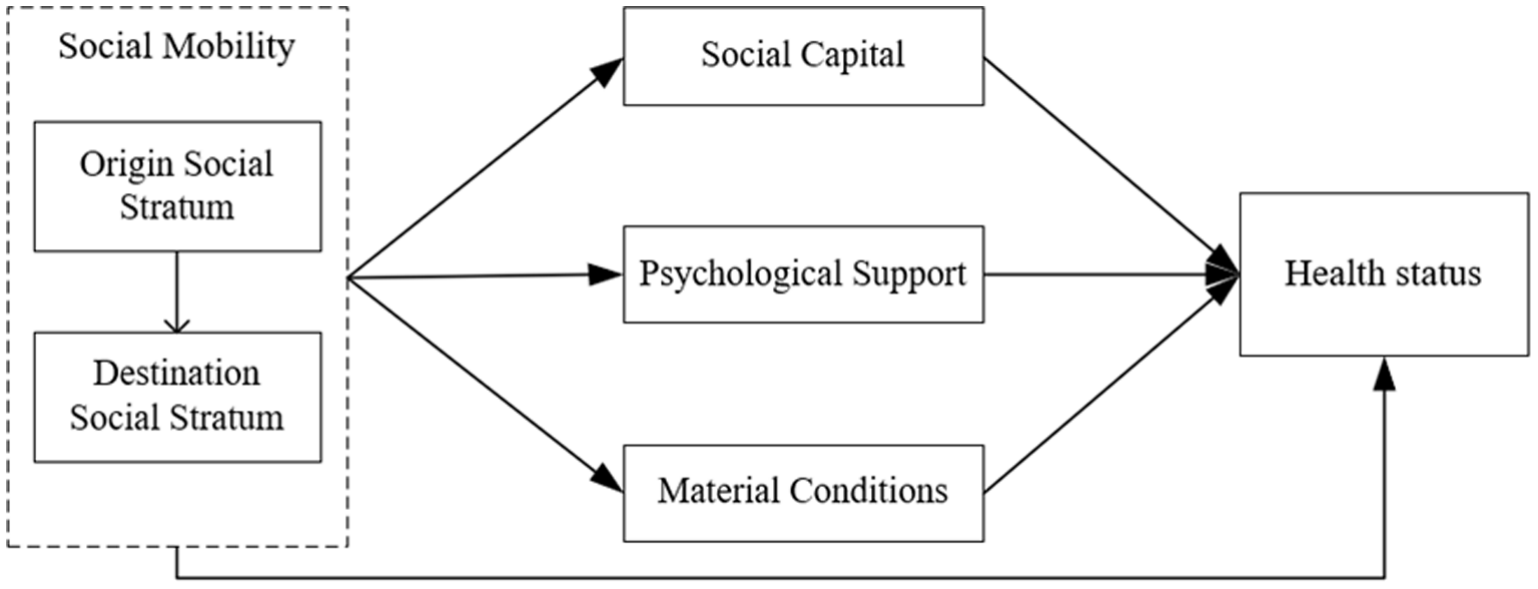
The theoretical analytical framework.

Although the impact of social mobility on health varies depending on the direction of mobility and the research perspective, most studies indicate a positive relationship between upward mobility and health outcomes. This relationship can be examined from three key dimensions: material conditions, psychological support, and social capital (Figure 1). In terms of material conditions, social mobility often accompanies improved living conditions, such as healthier dietary and more accessible healthcare, which provide material conditions for better health (32, 33). Psychologically, social mobility signifies self-improvement through overcoming socioeconomic barriers, which can produce positive psychological effects such as a sense of well-being and enhancement of personal self-confidence (20, 34). These psychological benefits support improvements in individual health. In the realm of social capital, individuals who experience upward mobility tend to retain some of their original social ties while gradually integrating into the social circles of higher social strata, thereby diversifying and expanding their social interactions. Furthermore, social mobility enhances individuals’ sense of identification with the prevailing social system (21), contributing to their overall health improvement through enhanced social capital (35).

Based on the above analysis, we propose the following hypothesis:

*H*1: Social mobility has a positive impact on health, contributing to improved health outcomes.

*H*2: Social mobility promotes healthier dietary and reduces unfulfilled healthcare needs, providing material conditions for health improvement.

*H*3: Social mobility enhances well-being and self-confidence, offering psychological support for health improvement.

*H*4: Social mobility fosters social interaction and social identity, providing social capital to improve health outcomes.

## 3 Research design

### 3.1 Dataset

The data for this study were derived from the 2021 Chinese General Social Survey (CGSS). The CGSS employs a multi-stage stratified sampling method, selecting the top five cities based on economic level, education level, and urban openness through factor analysis. These cities are designated as the mandatory layer, while all households outside the urban districts of these cities are considered the selected layer. The selected layer is divided into 50 strata, consisting of 19 district layers and 31 county layers. In the mandatory layer, streets are used as primary sampling units (PSUs), and residential committees serve as secondary sampling units (SSUs). In the selected layer, districts, county-level cities, and counties are used as PSUs, and residential or village committees are used as SSUs to select household units. Finally, one individual aged 18 or older is randomly selected from each chosen household as the final survey subject.

In the 2021 CGSS, a total of 8,148 samples were collected nationwide, encompassing 700 variables covering socio-demographic attributes, health, lifestyle, social attitudes, class identity, and other aspects. This broad coverage aids in controlling confounding factors in the analysis, ensuring the independence and accuracy of the impact of social mobility on health. As a result, the CGSS is widely used in academic research on social mobility and population health (36, 37). After excluding records with missing key variables, this study obtained 3,752 valid samples, including 2,172 rural household registrations and 1,580 urban household registrations, with male participants accounting for 47.25%. The average age of the sample was 51.987 years. All data analyses presented in this paper were conducted using Stata 17.

### 3.2 Variables

#### 3.2.1 Dependent variable

Self-rated health. Self-rated health represents an individual’s subjective perception and psychological appraisal of their health status, which significantly reflects an integrated assessment of one’s health and has demonstrated predictive capacity for both mortality and morbidity (38, 39), with high data accessibility. In this study, self-rated health is used to characterize the health status of the sample population, measured through the CGSS questionnaire item: “How do you feel about your current physical health status?” Responses are categorized into five levels: very unhealthy, somewhat unhealthy, average, somewhat healthy, and very healthy, coded numerically as 1 through 5, respectively.

#### 3.2.2 Primary independent variable

Upward social mobility. The CGSS questionnaire employs a visual analog scale accompanied by a ladder image, inviting respondents to self-assign their social class positioning at different points in time, ranging from 1 (lowest) to 10 (highest). Drawing from Jindong et al. (49), this study consolidates adjacent classes into five broader categories: very low, low, middle, high, and very high social classes. For the baseline regression analysis, a binary variable (0–1) is utilized to denote social mobility, coded as “1” if the current social class ranking is higher than that of 10 years prior, indicating upward social mobility, and “0” otherwise. In the subsequent advanced analysis aimed at exploring how the direction and magnitude of social class change affect health outcomes, the difference between an individual’s current social class and that of a decade ago is employed as a measure of social class transition.

#### 3.2.3 Control variables

This study also controls for factors that may give rise to variations in health status, chief among which are gender, age, age squared, Hukou type, marital status, educational attainment, employment status, logarithm of annual household income, number of children, smoking habits, alcohol consumption, participation in medical insurance, and geographic region. A detailed descriptive statistical summary of these variables is provided in Table 1.

**Table 1:** Variable definition and descriptive statistics.

Variable	Variable description	Frequency/Mean	Percentage/SD
Self-rated health	Very unhealthy	190	5.06
	Somewhat unhealthy	484	12.9
	Average	1,107	29.5
	Somewhat healthy	1,330	35.45
	Very healthy,	641	17.08
Upward social mobility	No	2,364	63.01
	Yes	1,388	36.99
Gender	Male	1,773	47.25
	Female	1,979	52.75
Age	Continuous variable	51.987	16.377
Age squared	Age ^2/100	29.708	16.993
Hukou type	Agricultural	2,172	57.89
	Non-agricultural	1,580	42.11
Marital status	Unmarried	886	23.61
	Married	2,866	76.39
Educational attainment	Illiteracy	367	9.78
	primary school and below	779	20.76
	Middle school	1,142	30.44
	High school	698	18.6
	College degree or above	766	20.42
Employment status	Not working	1,690	45.04
	Engaged in agricultural work	636	16.95
	Engage in non-agricultural work	1,426	38.01
Household income	Log(annual household income)	10.478	2.360
Number of children	Continuous variable	1.660	1.152
Smoking habits	Never smoked	2,489	66.34
	Quit	373	9.94
	Still have	890	23.72
Alcohol consumption	Never	2,457	65.49
	Sometimes	1,096	29.21
	Everyday	199	5.3
Medical insurance	Uninsured	176	4.69
	Basic medical insurance	3,027	80.68
	Commercial health insurance	38	1.01
	The two types of insurance above	511	13.62
Geographic region	Eastern Region	1,442	38.43
	Central Region	1,058	28.2
	Western Region	1,033	27.53
	Northeastern Region	219	5.84

For categorical variables, report the frequency counts and percentages; for continuous variables, report the mean and standard deviation.

### 3.3 Methods

#### 3.3.1 Ordinal multinomial logistic regression model

The primary independent variable in this study is self-rated health, which encompasses five ordinal categories: very unhealthy, somewhat unhealthy, average, somewhat healthy, and very healthy. Consequently, an ordinal logistic regression model is employed for analysis, given its suitability for handling dependent variables with ordered categories. The ordinal logistic regression equation utilized is formulated as follows:


(1) $$Y_{i}^{*}=\alpha X_{i}+\varepsilon_{i},i=1,2,\ldots ,5$$

The unobservable latent variable $Y_{i}^{*}$ has an underlying relationship with the observed ordinal outcome of self-rated health, which can be described as follows:


(2) $$Y_{i}=\left\{\begin{array}{c} 1,Y_{i}^{*}\leq \beta_{1}\\ 2,\beta_{1}<Y_{i}^{*}\leq \beta_{2}\\ \vdots \vdots \\ 5,Y_{i}^{*}>\beta_{4} \end{array}\right.$$

Where $\beta_{1},\beta_{2},\beta_{3},\beta_{4}$ represent the estimable parameter, known as the “cut-point” or threshold parameter, and $\beta_{1}<\beta_{2}<\beta_{3}<\beta_{4}$. The ordinal logistic regression model is specified as follows:


(3) $$\frac{lnp\left(y\leq j\right)}{p\left(y\geq j\right)}=y_{j}+\overset{n}{\underset{i=1}{\sum }}\delta_{i}x_{i},j=1,2,\ldots ,5$$

Let *j* represent the self-rated health levels, $y_{j}$ denote the intercept, $x_{i}$ be the factors influencing self-rated health, and $\delta_{i}$ be the regression coefficients for each influencing factor, representing the impact of the explanatory variables on the dependent variable.

#### 3.3.2 Karlson-Holm-Breen mediation analysis

Common methods for testing mediation effects, such as the “three-step method,” Sobel test, and Bootstrap test, are premised on the comparability of coefficients in linear regression models. However, when the dependent variable is non-continuous, scale issues render coefficients incomparable, thereby hindering the application of techniques for examining mediating variables in nonlinear regression frameworks. Therefore, this paper employs the KHB mediation analysis method proposed by Kohler, Karlson, and Holm to discuss how social mobility affects health outcomes. Essentially, this method analyzes the underlying linear model behind the nonlinear probability model, using the residual term from the regression of the mediator on the independent variable to replace the mediator itself. This approach eliminates the model scale changes caused by changes in the independent variable within the nonlinear probability model, thereby obtaining the proportion of the mediation effect.


(4) $$Y_{i}=\alpha +\beta_{1}MOB_{i}+\delta_{1}Z+\gamma_{1}X_{i}^{\prime }+\varepsilon_{2i}$$

(5) $$Y_{i}=\alpha^{\prime }+\beta_{1}^{\prime }MOB_{i}+\gamma_{2}X_{i}^{\prime }+\varepsilon_{2^{\prime }i}$$

Equation 4 represents the model where the mediator variable is controlled, whereas Equation 5 illustrates the model without controlling for the mediator. In these equations, $X_{i}^{\prime }$ signifies the control variables, and Z denotes the mediator variable. The indirect effect, denoted by $\Delta \beta =\beta_{1}^{\prime }-\beta_{1}$, captures how the independent variable operates through the mediator to influence the dependent variable. In the estimation of the baseline regression models, we obtain fitted coefficients $b_{1}$ and $b_{2}$, b1=β1σ1, b2=β1′σ2, with $\sigma_{1}$ and $\sigma_{2}$ serving as scale parameters that depend on the standard deviation of the residuals in respective models. Conventionally, scale coefficients may vary across distinct models; however, they adhere to the constraint that $b_{2}-b_{1}≅\beta_{1}^{\prime }-\beta_{1}$,

The KHB method initially treats the mediator variable *Z* as the dependent variable, regressing it on $Y_{i}$ as the independent variable, forming the model $Z=c+d\cdot Y_{i}+r$, where *r* represents the residual from this regression. Subsequently, this residual *r* is incorporated as an independent variable in a subsequent model fitting exercise, resulting in:


(6) $$Y_{i}^{*}=\alpha^{\prime }+{\beta \prime }_{1}^{*}MOB_{i}+\delta_{2}^{*}r+\gamma_{2}X_{i}^{\prime }+\varepsilon_{2}\prime_{i}^{*}$$

Equation 6 exhibits a comparable model fit to Equation 5, indicating that $\varepsilon_{2i}=\varepsilon_{2}\prime_{i}^{*}$, suggesting that $Y_{i}$ and r are not perfectly correlated. Consequently, the indirect effect of variable Z in the baseline model can be represented as follows: b2∗−b1=β1′σ2∗−β1σ1. Thus, the direct effect, indirect effect, and total effect of the independent variable $MOB_{i}$ on the dependent variable $Y_{i}$ in the baseline model are disentangled, and by comparing the ratio of the indirect effect to the total effect, the magnitude of the mediator variable *Z*’s influence is quantified.

## 4 Results

### 4.1 Health effects of social mobility

Table 2 presents the mean self-rated health status and sample distribution segmented by individuals’ origin and destination social classes. The diagonal cells display the self-rated health status and sample distribution of individuals who experienced no social mobility. In contrast, the cells above and below the diagonal represent individuals who experienced upward and downward social mobility, respectively. Among the group with no social mobility, the overall self-rated health level gradually increased from very low to high socioeconomic status, with a slight decline at very high economic status. Yet, it remains above the average of the total sample. The mean self-rated health in the cells above the diagonal is generally higher than those below the diagonal, indicating that individuals who experienced upward social mobility tend to have better health status than those who experienced downward social mobility.

**Table 2:** Social stratum transition matrix and self-assessed health status cross-tabulation.

Origin social stratum	Destination social stratum					
	Very low	Low	Middle	High	Very high	Total
Very low	3.05 (517)	3.51 (387)	3.57 (192)	4.00 (7)	2.33 (9)	3.30 (1112)
Low	2.65 (71)	3.49 (566)	3.70 (616)	3.53 (36)	2.50 (2)	3.54 (1291)
Middle	2.64 (44)	3.24 (141)	3.56 (736)	3.92 (106)	3.78 (18)	3.52 (1045)
High	2.43 (7)	3.43 (28)	3.50 (94)	3.85 (92)	4.07 (15)	3.63 (236)
Very high	2.89 (9)	2.67 (3)	3.33 (15)	3.40 (15)	3.54 (26)	3.34 (68)
Total	2.97 (648)	3.46 (1125)	3.61 (1653)	3.81 (256)	3.53 (70)	3.47 (3752)

Note: The numbers in the cells represent the sample mean of self-rated health status, and the values in parentheses indicate the sample size. The shaded area denotes the boundary for social mobility, entries above this area indicate upward social mobility, while those below indicate downward social mobility.

**Figure 2:**
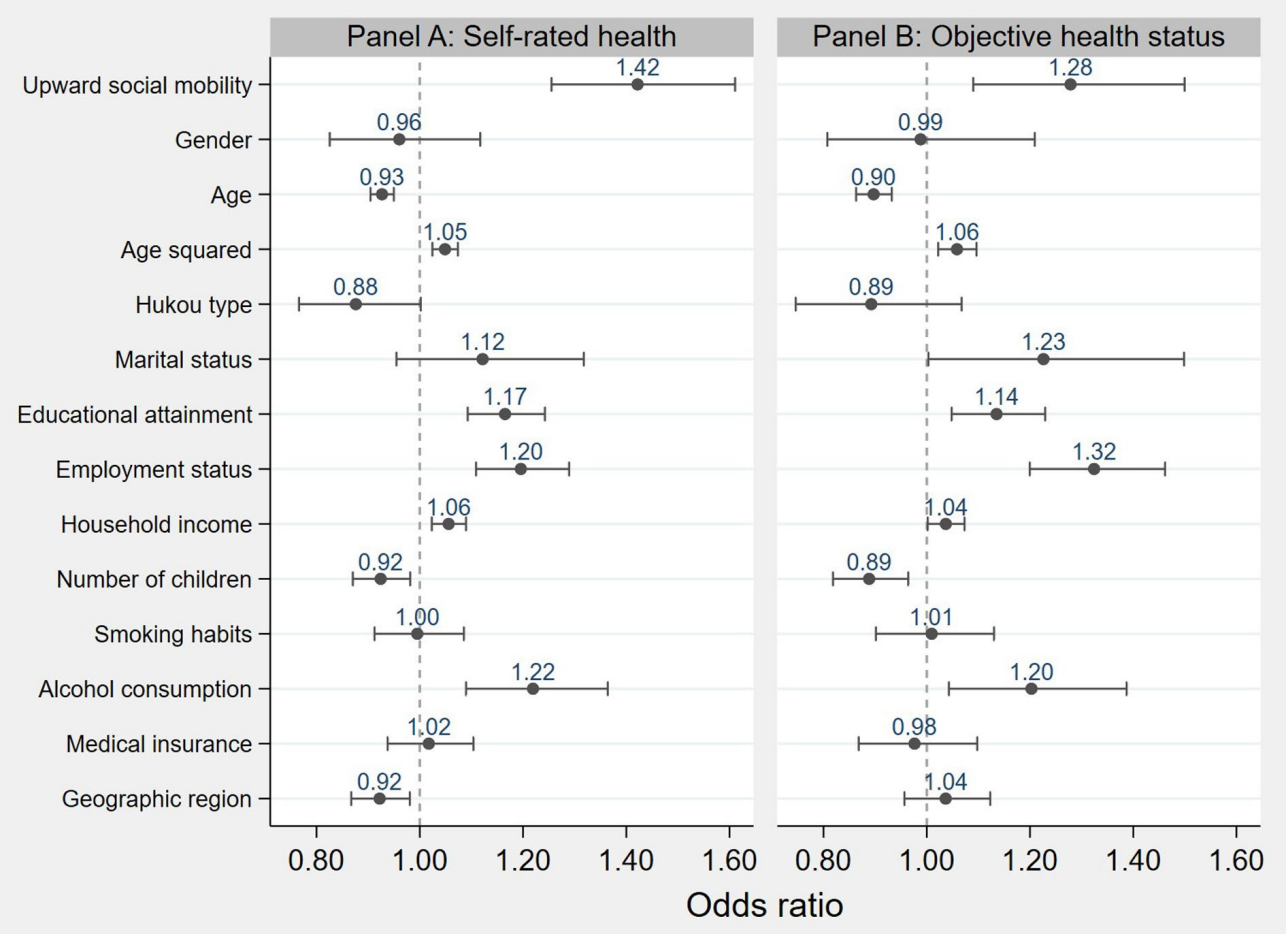
The impact of social mobility on health status. Note: Solid dots indicate the odds ratios, and short lines represent the 95% confidence intervals.

Panel A in Figure 2 illustrates the impact of social mobility on self-rated health status as estimated by an ordered multinomial logistic model. The regression results indicate that social mobility has a significant positive effect on self-rated health, with an odds ratio (OR) of 1.422 (*p* = 0.000). This implies that, holding other factors constant, individuals who experience social mobility are 1.422 times (e^0.352) more likely to report better self-rated health compared to those who do not. In other words, individuals who undergo social mobility are more likely to have better self-rated health than their counterparts. These findings suggest a clear association between social mobility and individual health, with social mobility exerting a positive influence on self-rated health status.

Given the limitations of subjective health indicators and the diversity of health measurement methods, this study employs objective health indicators to conduct robustness checks. Specifically, based on whether respondents reported “having a chronic illness or long-term health problem,” individuals with such conditions are defined as unhealthy and assigned a value of 0. Conversely, those without chronic illness or long-term health issues are considered to be in good health and assigned a value of 1. The results are presented in Panel B of Figure 2, which shows that social mobility has a significant positive effect on objective health (OR = 1.278, *p* = 0.003), consistent with the baseline regression results.

### 4.2 The solution to endogenous problems

Although the previous sections confirmed a significant positive correlation between social mobility and self-rated health status, endogeneity issues may still threaten conclusions due to random factors, omitted variables, and bidirectional causality. We employed placebo tests, propensity score matching, and the instrumental variable method to address these concerns and mitigate endogeneity issues.

#### 4.2.1 Placebo tests

**Figure 3:**
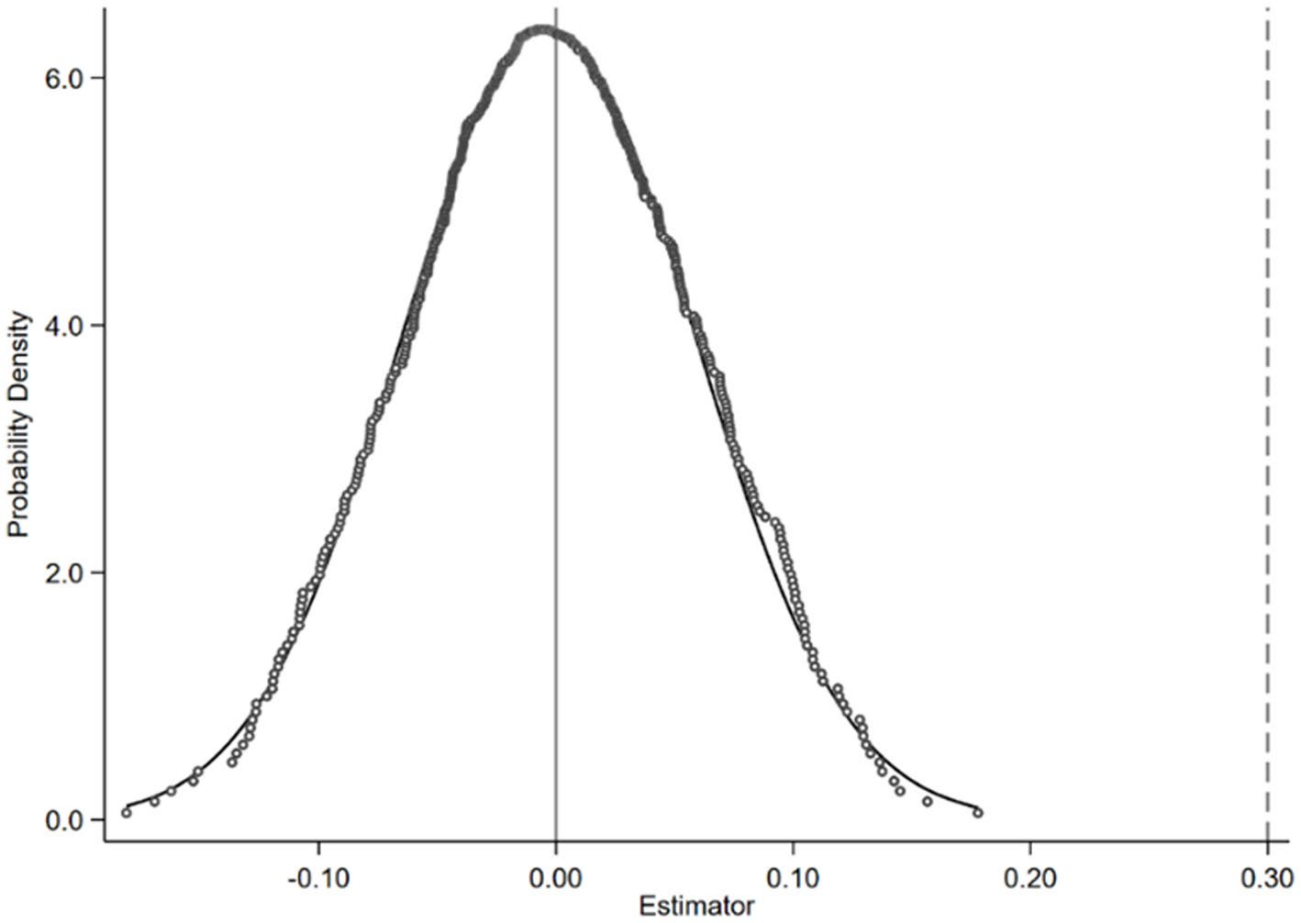
Placebo tests.

To exclude the potential impact of omitted variables and random factors on the aforementioned research findings, drawing upon the studies of Yuhui, Huan (40) and La Ferrara, Chong (41), this paper conducts placebo tests by constructing fictitious core explanatory variables. The essence of this approach lies in substituting the original explanatory variable with a fabricated dummy variable that theoretically should exert no influence on the dependent variable. If the dummy variable influences the dependent variable, it suggests the presence of omitted variable bias in the baseline regression results. Accordingly, we randomly generate the states of sample’s social mobility and refitted Model (1) to compute the model coefficients. This study employs a Monte Carlo test to repeat the above steps 500 times. The purpose of multiple simulations is eliminate the possibility of randomness in the results, and exclude any systematic biases by comparing the consistency of different runs, thereby obtaining a more stable and reliable distribution of results. We then analyze the regression coefficients of social mobility from the 500 regressions, generate a kernel density plot, and compare them with the coefficients of social mobility in Model (1). As shown in Figure 3, the estimated coefficients obtained from the pseudo-explanatory variables are concentrated around 0 and follow a normal distribution, all of which are much smaller than the true estimated coefficient (0.352) in Model (1). This indicates that fabricated social mobility scenarios cannot support a positive correlation between social mobility and self-rated health, suggesting that our regression model can effectively control for omitted variable bias.

#### 4.2.2 Propensity score matching

To mitigate the impact of sample selection biases and inter-group heterogeneity on the research outcomes, this study employs the Propensity Score Matching (PSM) technique. The fundamental rationale behind PSM is to identify a counterpart individual “*j*” in the control group whose observable characteristics closely resemble those of an individual “*i*” in the treatment group, thereby establishing a counterfactual scenario that facilitates estimation of the treatment effect for individual “*i*.” Employing neighbor matching, radius matching, kernel matching, spline matching, and Mahalanobis matching, we found that social mobility positively affects self-rated health. The net effect of social mobility on self-rated health ranges between 16.1 and 20.2% (Table 3). The PSM analysis outcomes affirm that the positive association between social mobility and self-assessed health remains robust even after adjusting for sample selection biases.

**Table 3:** The processing effect of propensity score matching.

Matching method	Health outcomes in middle and old age				
	T	C	ATT	S.E.	T-stat
Neighbor matching	3.624	3.463	0.161***	0.035	4.55
Radius matching	3.638	3.444	0.194***	0.036	5.35
Kernel matching	3.638	3.447	0.191***	0.036	5.27
Spline matching	3.638	3.459	0.179***	0.028	6.31
Mahalanobis matching	3.638	3.435	0.202***	0.037	5.55

***t > 2.58, **t > 1.96, *t > 1.64.

#### 4.2.3 Instrumental variable estimation

On one hand, higher social classes typically have greater access to health resources, contributing to better health. On the other hand, good health can enhance an individual’s work efficiency and learning ability, increasing their chances of social mobility. To tackle this potential endogeneity issue where the explanatory and response variables may be mutually causal, this study employs the instrumental variable (IV) method. Following Bentolila, Michelacci (42), we adopt aggregate-level data as an instrument for the lower-level variable, specifically utilizing provincial upward social mobility rates as the instrumental variable.

Table 4 presents the regression results using the Instrumental Variables Two-Stage Least Squares (IV-2SLS) method. As shown in Model (3), the provincial upward social mobility rate has a coefficient of 0.955 on the sample individuals’ social mobility, statistically significant at the 1% level, strongly validating the aforesaid argument about the instrument’s relevance. This confirms that provincial upward social mobility rates positively influence individuals’ ability to achieve social class advancement. In Model (4), the coefficient of social mobility on self-rated health remains positive, suggesting that, after addressing potential endogeneity issues with the IV method, the positive effect of social mobility on self-assessed health found in the baseline regression persists. Additionally, the Cragg-Donald Wald F statistic and the Kleibergen-Paap Wald rk F statistic, both used to test for weak instruments, exceed the critical value associated with a 10% maximal IV size (16.38), demonstrating that this study does not suffer from identification or weak instrument problems.

**Table 4:** The results of IV-2SLS.

Variable	IV-2SLS	
	(3)	(4)
Dependent Variable	Upward social mobility	Self-rated Health
	Phase 1	Phase 2
Provincial upward social mobility rates	0.955***	
	(0.146)	
Upward social mobility		0.737**
		(0.322)
Individual Characteristics	Yes	Yes
Household Characteristics	Yes	Yes
Social Security Characteristics	Yes	Yes
Health Behavior Characteristics	Yes	Yes
Geographical Characteristics	Yes	Yes
Cragg-Donald Wald F statistic		41.703
Kleibergen-Paap rk LM		41.711 0.000
Observations	3,752	3,752

***p < 0.01, **p < 0.05, *p < 0.1.

### 4.3 Heterogeneity analysis

#### 4.3.1 Heterogeneity analysis based on gender

Significant differences between men and women in physiological, psychological, and behavioral aspects may influence the generation and manifestation of health effects. An analysis of gender disparities in how social mobility impacts self-rated health reveals that the coefficient for the effect of social mobility on men’s self-rated health is 0.462 (*p* = 0.000), whereas the corresponding coefficient for women is substantially lower at 0.273 (*p* = 0.002), indicating that social mobility exerts a much stronger positive influence on men’s self-perceived health compared to women’s. The difference in the coefficient of influence between the two groups is 0.189, which passes the Fisher combination test at the 10% level (Table 5).

**Table 5:** Heterogeneity analysis.

Variable	Gender		Early socioeconomic status	
	Male	Female	Low	High
Upward social mobility	0.462*** (−4.97)	0.273*** (−3.10)	0.465*** (−5.80)	0.267** (−2.48)
Permutation test	0.189*		0.199**	
Control variable	Yes	Yes	Yes	Yes
Observations	1,773	1,979	2,217	1,535
R	0.058	0.069	0.064	0.056

***p < 0.01, **p < 0.05, *p < 0.1.

#### 4.3.2 Heterogeneity analysis based on early socioeconomic status

This study utilizes the social class of the respondent’s family at age 14 to represent early socioeconomic status. Samples are divided into two groups—high and low early socioeconomic status—based on whether their family’s social class at age 14 was above or below the median. Heterogeneity analysis results based on early socioeconomic status indicate that social mobility positively impacts self-rated health outcomes for groups with higher and lower early socioeconomic status. Particularly noteworthy is that for individuals with lower early socioeconomic status, the effect of social mobility on self-rated health is markedly more substantial. The difference in the effect sizes between the two groups is 0.199, passing the permutation test at the 5% significance level (Table 5). Therefore, the health-promoting effect of social mobility is more pronounced among those with lower early socioeconomic status.

### 4.4 Mechanism analysis

From our theoretical analysis, the mechanisms by which social mobility influences health include the social capital mechanism, psychological support mechanism, and material conditions mechanism. Drawing from the methodologies of Weitong and Jiayin (43), we augment our baseline regression with mediators corresponding to each of these mechanisms, utilizing the changes in the coefficient estimates of the independent variable—social mobility—to ascertain the validity of the mediating mechanisms and the precise transmission channels. If the coefficient of the dependent variable decreases after introducing a single mediator, it indicates that the mediator serves as a positive transmission mechanism through which social class mobility affects self-rated health. Conversely, if the coefficient of the dependent variable increases after introducing the mediator, it suggests that the mediator represents a negative transmission mechanism between the independent and dependent variables. Additionally, considering that coefficients in nonlinear probability models are not comparable due to scale issues, making it difficult to assess the size of each variable’s mediating effect accurately, this study further employs the KHB mediation analysis method to analyze the strength of each variable’s mediation effect.

#### 4.4.1 Material conditions mechanism

This study employs the frequency of consuming fresh fruits or vegetables and the occurrence of preceding necessary medical care due to affordability within the past year to evaluate residents’ material living conditions from the perspectives of dietary intake and potential healthcare demand. According to the results from Model (5) in Table 6, after introducing dietary intake as a mediator in the baseline model, the regression coefficient of the primary explanatory variable—social mobility—significantly diminishes to 0.331 (*p* = 0.000), while the coefficient for dietary intake stands at 0.290 (*p* = 0.000). Additionally, referring to the data in Table 7, the percentage of confounding effects attributable to dietary intake is 7.32%. This indicates that the enhancement in self-rated health following social mobility is partially attributable to improved intake of fresh fruits and vegetables post-mobility, accounting for 7.32% of the health effect.

**Table 6:** The mechanism of social mobility affecting health.

Variable	(5)	(6)	(7)	(8)	(9)	(10)
	Dietary	Healthcare	Well-being	Self-confidence	Social interaction	Social identity
Upward social mobility	0.331***	0.345***	0.253***	0.323***	0.338***	0.320***
	(0.092)	(0.109)	(0.065)	(0.093)	(0.064)	(0.064)
Gender	−0.046	−0.002	−0.047	0.060	−0.059	−0.012
	(0.114)	(0.134)	(0.078)	(0.117)	(0.077)	(0.078)
Age	−0.069***	−0.068***	−0.055***	−0.087***	−0.073***	−0.073***
	(0.018)	(0.020)	(0.013)	(0.018)	(0.012)	(0.013)
Age squared	0.040**	0.037*	0.022*	0.052***	0.045***	0.043***
	(0.017)	(0.020)	(0.012)	(0.018)	(0.012)	(0.012)
Hukou type	−0.178*	−0.105	−0.140**	−0.159	−0.115*	−0.121*
	(0.095)	(0.112)	(0.070)	(0.097)	(0.069)	(0.069)
Marital status	0.108	0.127	0.001	0.027	0.123	0.107
	(0.120)	(0.133)	(0.083)	(0.118)	(0.082)	(0.083)
Educational attainment	0.187***	0.164***	0.113***	0.142***	0.147***	0.146***
	(0.046)	(0.053)	(0.033)	(0.046)	(0.033)	(0.033)
Employment status	0.198***	0.173***	0.156***	0.198***	0.182***	0.169***
	(0.054)	(0.066)	(0.039)	(0.054)	(0.039)	(0.039)
Household income	0.053**	0.072***	0.041***	0.053**	0.054***	0.053***
	(0.024)	(0.026)	(0.015)	(0.024)	(0.016)	(0.016)
Number of children	−0.060	−0.009	−0.092***	−0.037	−0.076**	−0.085***
	(0.050)	(0.061)	(0.031)	(0.050)	(0.031)	(0.031)
Smoking habits	0.023	0.035	−0.011	−0.006	−0.011	0.007
	(0.065)	(0.080)	(0.045)	(0.067)	(0.044)	(0.044)
Alcohol consumption	0.137	0.013	0.221***	0.168*	0.183***	0.201***
	(0.090)	(0.109)	(0.059)	(0.095)	(0.058)	(0.058)
Medical insurance	−0.015	−0.094	−0.023	−0.001	0.006	0.019
	(0.059)	(0.072)	(0.043)	(0.061)	(0.042)	(0.042)
Geographic region	−0.115***	−0.093*	−0.069**	−0.112**	−0.078**	−0.092***
	(0.044)	(0.054)	(0.032)	(0.045)	(0.031)	(0.032)
Dietary	0.290***					
	(0.061)					
Healthcare		−1.194***				
		(0.165)				
Well-being			0.593***			
			(0.045)			
Self-confidence				0.551***		
				(0.052)		
Social interaction					0.128***	
					(0.030)	
Social identify						0.204***
						(0.035)
/cut1	−3.574***	−4.705***	−2.762***	−3.214***	−4.527***	−4.205***
	(0.600)	(0.661)	(0.409)	(0.577)	(0.393)	(0.403)
/cut2	−2.024***	−3.036***	−1.192***	−1.618***	−3.010***	−2.689***
	(0.594)	(0.653)	(0.408)	(0.574)	(0.389)	(0.400)
/cut3	−0.376	−1.317**	0.478	0.107	−1.397***	−1.073***
	(0.594)	(0.648)	(0.409)	(0.574)	(0.388)	(0.399)
/cut4	1.504**	0.639	2.431***	2.040***	0.496	0.831**
	(0.593)	(0.642)	(0.409)	(0.573)	(0.386)	(0.397)
Observations	1,851	1,363	3,750	1,834	3,752	3,735
Pseudo R^2^	0.071	0.089	0.083	0.089	0.064	0.066
Log-likelihood	−2488.199	−1817.586	−4969.610	−2414.016	−5072.050	−5038.825

Robust standard errors in parentheses. ***p < 0.01, **p < 0.05, *p < 0.1.

**Table 7:** The results of KHB.

Effect type	Social capital mechanism		Psychological support mechanism		Material conditions mechanism	
	Dietary	Healthcare	Well-being	Self-confidence	Social interaction	Social identity
Total effect	0.357***	0.391***	0.377***	0.385***	0.356***	0.355***
	0.090	0.105	0.063	0.091	0.063	0.063
Direct effect	0.331***	0.345***	0.253***	0.323***	0.338***	0.320***
	0.090	0.105	0.064	0.091	0.063	0.063
Indirect effect	0.026**	0.046**	0.124***	0.062**	0.017***	0.036***
	0.012	0.022	0.018	0.025	0.006	0.009
Confounding ratios	7.32%	11.67%	32.99%	16.18%	4.86%	10.04%

***p < 0.01, **p < 0.05, *p < 0.1.

**Figure 4:**
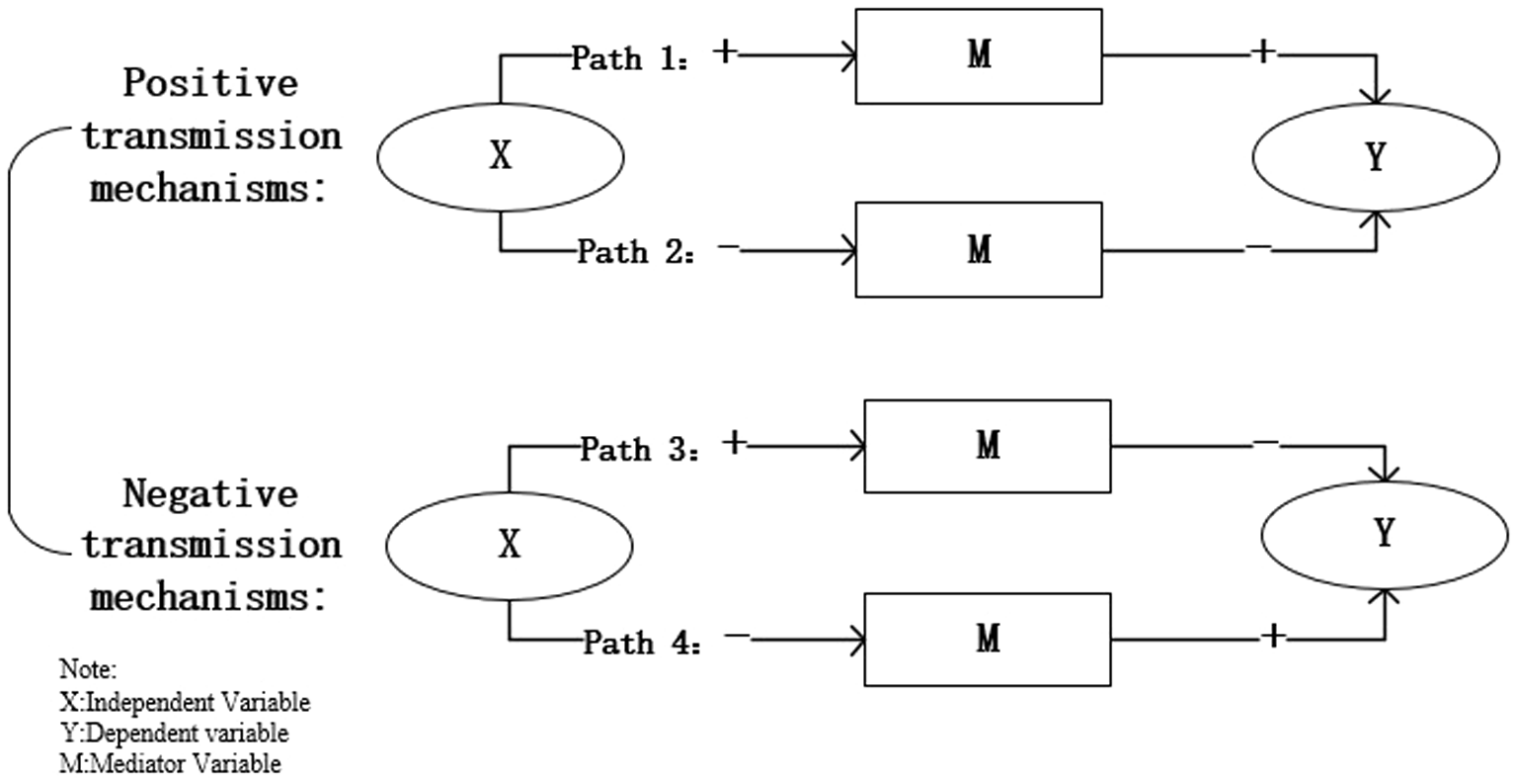
Transmission mechanism of the mediating effect.

Upon introducing the latent healthcare demand variable into the baseline regression, the impact coefficient of social mobility reduces to 0.345 (*p* = 0.002). Unlike all other mediators, the coefficient for the latent healthcare demand mediator becomes negative, at −1.194 (*p* = 0.000), with the corresponding transmission mechanism depicted in Pathway 2 of Figure 4. Hypothesis H2 is thereby substantiated.

#### 4.4.2 Psychological support mechanism

This study uses well-being and self-confidence as proxy variables to test the mediating role of psychological support mechanisms in the relationship between social mobility and self-rated health. Well-being is derived from residents’ self-assessment of their happiness, while self-confidence is measured by whether residents have ever lost self-confidence. The results support hypothesis H3, corresponding to mediation pathway 1. Well-being and self-confidence are positive mechanisms through which social mobility affects self-rated health, explaining 32.99 and 16.18% of the total effect, respectively. Among all the mediating variables, well-being has the strongest explanatory power.

#### 4.4.3 Social capital mechanism

Drawing from the methodology of Fuqin and Yuyin (44), this study employs social interaction and social identify to gage the sample’s social capital. Social interaction is measured by the frequency of socializing or visiting others during leisure time, categorized into five levels from “never” to “very frequently.” Social identity is derived from the respondent’s perception of societal fairness, ranging from “completely unfair” to “completely fair” across five categories. As demonstrated in Table 6, Models (9) and Models (10) present the mediating effects of social interaction and social identify, respectively.

Firstly, the coefficients for the impact of social interaction and social identify are significantly positive at the 1% level, suggesting that closer social ties and higher degrees of social identify can positively influence self-rated health to a certain extent. The coefficients for the effect of social mobility stand at 0.338 and 0.330, respectively, and are statistically significant at the 1% level. Compared to the coefficient for the dependent variable in the baseline regression (0.352), these figures show a decline, indicating that social interaction and social identify serve as mechanisms through which social mobility positively affects self-rated health status. In summary, the social capital mechanism by which social mobility impacts self-rated health is illustrated in Pathway 1 of Figure 4, showing that social class transitions improve self-rated health by increasing social interaction and enhancing social identify. Hypothesis H4 is thus supported. According to the KHB mediation effect analysis results in Table 7, social interaction and social identify account for 4.86 and 10.04% of the total effect, respectively, with social identify exhibiting a stronger mediating role than social interaction.

## 5 Discussion

### 5.1 Main conclusion

Based on the 2021 CGSS dataset, this study combines Ordered Logistic Regression, Instrumental Variables methods, and KHB mediation analysis to explore the health effects underlying social mobility and their mechanisms. Key findings include:

Firstly, a pronounced positive association exists between social mobility and individual health status, with upward social mobility positively influencing self-rated health. This conclusion aligns with the findings of Lei and Xing (45), which posited that upward social mobility contributes to improved self-rated health. This supports the rising from rags hypothesis (19). The research conclusion remains robust after addressing endogeneity concerns through placebo tests, propensity score matching, and instrumental variable methods.

Secondly, the positive impact of social mobility on self-rated health is greater for men than women. This finding can be explained by the theory of “intra-personal causal attribution” in social psychology, which posits that men and women differ in how they assess the primary causes determining the successes and failures in their lives. Men are more likely than women to attribute failure to factors beyond their control and success (i.e., upward social mobility) to their talents, abilities, and efforts (46). Consequently, once men experience upward social mobility, they are more likely to believe that they have succeeded due to their merits and efforts, which in turn may be more beneficial to their health outcomes (47).

Thirdly, social mobility positively impacts self-rated health outcomes for high and low initial SES groups. Still, the effect is stronger for the lower SES group. The underlying reason is that individuals from lower SES backgrounds have a stronger desire for social mobility and are more sensitive to changes in socioeconomic status. When their socioeconomic status rises, the health benefits are more pronounced (48). Additionally, compared to those with higher SES, individuals with lower SES face greater health risks and have more room for improvement in their health status. The marginal utility of social mobility is higher for this group, leading to more significant improvements in their health outcomes.

Fourthly, social class transitions impact residents’ health through mechanisms of social capital, psychological support, and material conditions. Within the social capital mechanism, close social interactions and strong social identity are conducive to enhancing residents’ health levels during upward social mobility. In the psychological support mechanism, the elevation of happiness and confidence constitutes a crucial pathway by which social mobility improves health status. Under the material conditions mechanism, upward social mobility furnishes the material groundwork for healthy dietary intake and reducing unfulfilled healthcare needs, prerequisites for improving residents’ health conditions.

### 5.2 Policy implication

Drawing from the findings of this study, aimed at addressing health stratification and narrowing the health disparities between social classes, the following policy recommendations are proposed:

Firstly, social mobility and health promotion should be strengthened. The government should implement comprehensive measures to promote social mobility and provide equal opportunities for social advancement to all individuals. By offering support through education, vocational training, and employment opportunities, more people can achieve upward mobility, thereby improving the overall health of the population. Additionally, efforts should be made to reduce welfare disparities between social classes, lowering the social costs associated with illness and promoting equal health opportunities.

Secondly, the government should address gender differences by designing targeted interventions. Our study finds that men benefit more than women from social mobility in terms of health. To promote health equity, social development should prioritize greater protection for women. Specifically, gender-equal employment policies should be implemented, advocating for fair pay and promotion opportunities to ensure women have equal access to job opportunities and treatment in the workplace. Additionally, more flexible and sustainable family support policies should be established, such as parental leave and family care leave, to alleviate the pressures women face in balancing career development and family responsibilities, thus providing them with better career advancement opportunities.

Thirdly, governments should focus on individuals with lower early-life socioeconomic status. Our research suggests that the positive impact of social mobility on health is more significant among individuals with lower socioeconomic status in the early stages. For low-income groups, it is essential to improve income distribution mechanisms, reform tax policies to reduce the tax burden on lower-income individuals, and increase taxes on higher-income earners to facilitate income redistribution and narrow the wealth gap. Moreover, a robust labor law framework should be established, promoting equal pay for equal work, setting minimum wage standards, and creating mechanisms for wage growth to ensure fair compensation and help individuals improve their income levels.

Fourthly, build a support system that encompasses material security, mental health services, and social capital to maximize the positive health impacts of social mobility. In terms of material security, the government should establish a comprehensive and high-level social security system to adequately meet basic needs such as food and healthcare, thereby improving the quality of life for the population. For mental health services, efforts should be made to promote mental health education, raise awareness, and establish a well-developed counseling service network that provides accessible psychological support. Moreover, the training of mental health professionals should be strengthened to enhance the quality of services. In the realm of community support, the government should invest in building community service facilities, providing convenient social spaces and activities to ensure all members can equally participate in social events. Additionally, community organizations should be encouraged and supported to strengthen connections and a sense of belonging among members, thereby enhancing social identity and cohesion.

### 5.3 Limitations

This study has several limitations. First, while we have identified a positive relationship between social mobility and health outcomes, other confounding factors, such as cultural background, genetic predispositions, and pre-existing health conditions, were not fully accounted for. These should be explored in future research. Second, the dependent variable’s ability to capture health status is constrained, relying on a single self-rated item prone to biases like recall and social expectation bias. Future studies could incorporate validated scales, such as SF-36 or EQ-5D, for a more comprehensive assessment. Third, the cross-sectional nature of the data limits our ability to establish causal relationships and mediation effects. Future research should use longitudinal data to better understand the temporal dynamics of these relationships. Finally, China’s unique institutional features, like the Hukou system, affect access to healthcare and education, potentially limiting the generalizability of our findings to other regions. Comparative studies across countries or regions with differing policies and cultural norms are needed to explore how these factors shape the link between social mobility and health outcomes.

## Data Availability

The datasets presented in this study can be found in online repositories. The names of the repository/repositories and accession number(s) can be found at: http://www.cnsda.org/index.php?r=projects/view&id=65635422.

## Footnotes

^1^“Pro-rich” and “pro-urban” respectively denote better health outcomes for wealthier and urban populations.
